# Supplementary material for: Determinants of Lipid Parameters in Patients without Diagnosed Cardiovascular Disease—Results of the Polish Arm of the EUROASPIRE V Survey
Source: J Clin Med. 2023 Apr 6;12(7):2738. doi: 10.3390/jcm12072738 (PMC10095493; doi:10.3390/jcm12072738)
Supplement: Supplementary file 1 [file jcm-12-02738-s001.zip › jcm-2285685-supplementary.pdf]

Journal of
Clinical Medicine

MDPI

Supplementary Materials

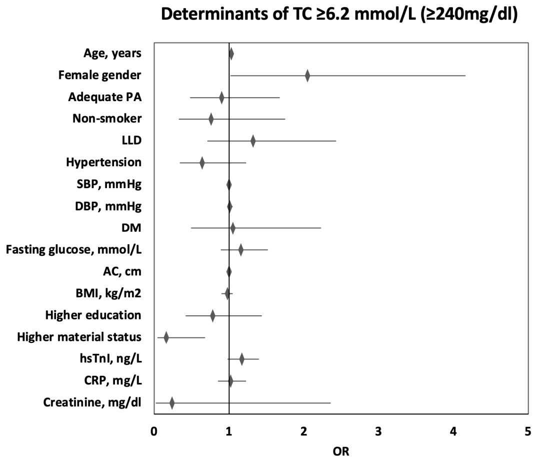

	Odds ratio	[95% CI]	p-value
Age, years	1.03	[1.01-1.06]	0.01
Female gender	2.05	[1.02-4.16]	0.05
Adequate PA	0.90	[0.48-1.68]	0.73
Non-smoker	0.76	[0.33-1.75]	0.53
LLD	1.32	[0.71-2.43]	0.38
Hypertension	0.64	[0.34-1.23]	0.18
SBP, mmHg	1.00	[0.98-1.02]	0.93
DBP, mmHg	1.01	[0.98-1.05]	0.45
DM	1.05	[0.49-2.23]	0.90
Fasting glucose, mmol/L	1.16	[0.89-1.52]	0.28
AC, cm	1.00	[0.97-1.02]	0.76
BMI, kg/m2	0.98	[0.90-1.05]	0.52
Higher education	0.78	[0.42-1.44]	0.42
Higher material status	0.16	[0.04-0.68]	0.01
hsTnI, ng/L	1.17	[0.98-1.40]	0.08
CRP, mg/L	1.02	[0.85-1.23]	0.85
Creatinine, mg/dl	0.24	[0.02-2.36]	0.22

Figure S1. Determinants of TC ≥6.2 mmol/L (≥240 mg/dl) based on the results of the univariate analysis.

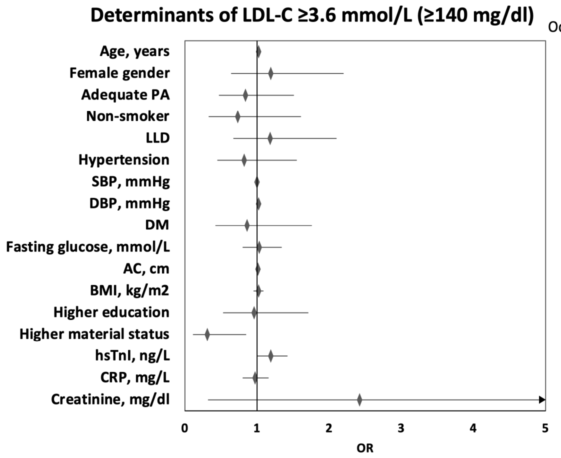

	Odds ratio	[95% CI]	p-value
Age, years	1.02	[1.00-1.05]	0.04
Female gender	1.19	[0.64-2.20]	0.58
Adequate PA	0.84	[0.47-1.51]	0.56
Non-smoker	0.73	[0.33-1.61]	0.44
LLD	1.18	[0.67-2.10]	0.57
Hypertension	0.82	[0.45-1.55]	0.57
SBP, mmHg	1.00	[0.98-1.02]	0.87
DBP, mmHg	1.02	[0.99-1.05]	0.20
DM	0.86	[0.42-1.76]	0.67
Fasting glucose, mmol/L	1.03	[0.80-1.34]	0.33
AC, cm	1.01	[0.99-1.03]	0.44
BMI, kg/m2	1.02	[0.95-1.09]	0.64
Higher education	0.96	[0.53-1.71]	0.88
Higher material status	0.31	[0.11-0.85]	0.02
hsTnI, ng/L	1.19	[1.00-1.42]	0.05
CRP, mg/L	0.97	[0.80-1.16]	0.71
Creatinine, mg/dl	2.42	[0.32-18.38]	0.39

Figure S2. Determinants of LDL-C ≥3.6 mmol/L (≥140 mg/dl) based on the results of the univariate analysis.

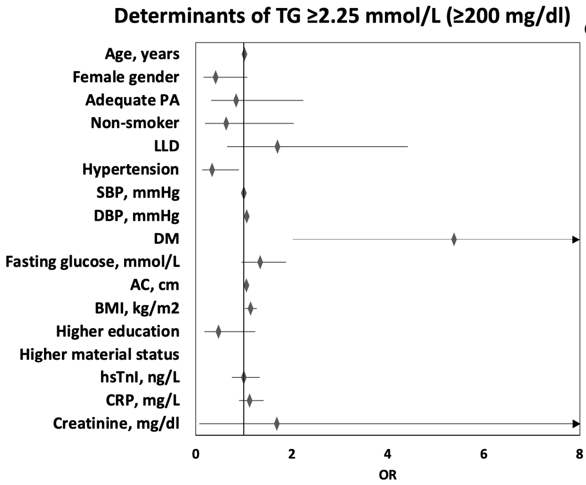

	Odds ratio	[95% CI]	p-value
Age, years	1.01	[0.98-1.05]	0.49
Female gender	0.41	[0.16-1.07]	0.07
Adequate PA	0.84	[0.32-2.24]	0.73
Non-smoker	0.63	[0.19-2.04]	0.44
LLD	1.70	[0.65-4.42]	0.28
Hypertension	0.34	[0.13-0.90]	0.03
SBP, mmHg	1.00	[0.97-1.03]	0.94
DBP, mmHg	1.06	[1.00-1.11]	0.03
DM	5.38	[2.02-14.33]	<0.001
Fasting glucose, mmol/L	1.34	[0.95-1.88]	0.10
AC, cm	1.05	[1.01-1.09]	0.01
BMI, kg/m2	1.14	[1.02-1.27]	0.03
Higher education	0.47	[0.18-1.24]	0.13
Higher material status	NA	NA	NA
hsTnI, ng/L	1.00	[0.75-1.33]	1.00
CRP, mg/L	1.12	[0.90-1.41]	0.32
Creatinine, mg/dl	1.69	[0.07-43.27]	0.75

Figure S3. Determinants of TG ≥2.25 mmol/L (≥200 mg/dl) based on the results of the univariate analysis.

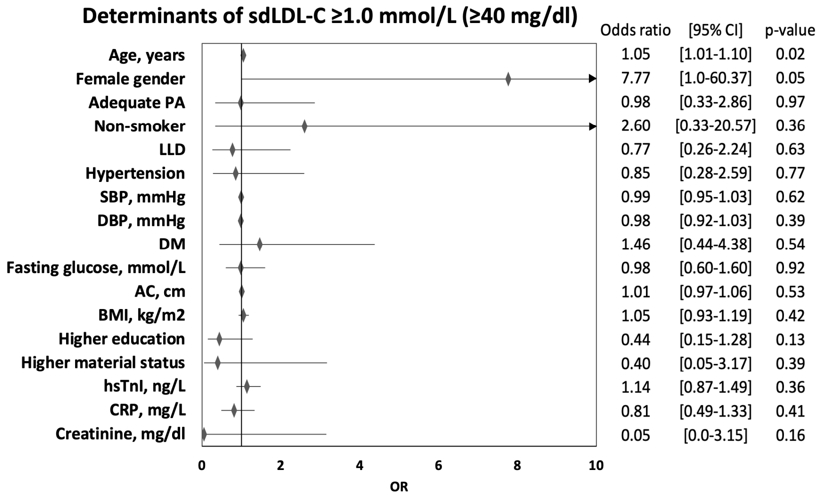

Figure S4. Determinants of sdLDL-C ≥1.0 mmol/L (≥40 mg/dl) based on the results of the univariate analysis.

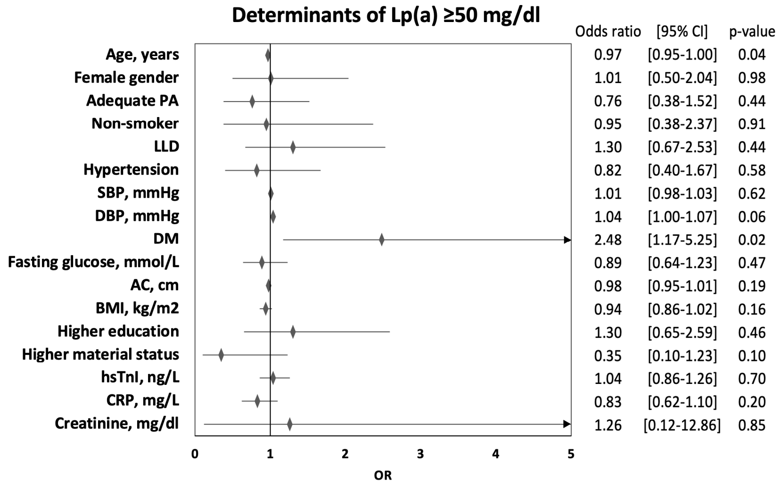

Figure S5. Determinants of Lp(a) ≥50 mg/dl based on the results of the univariate analysis.

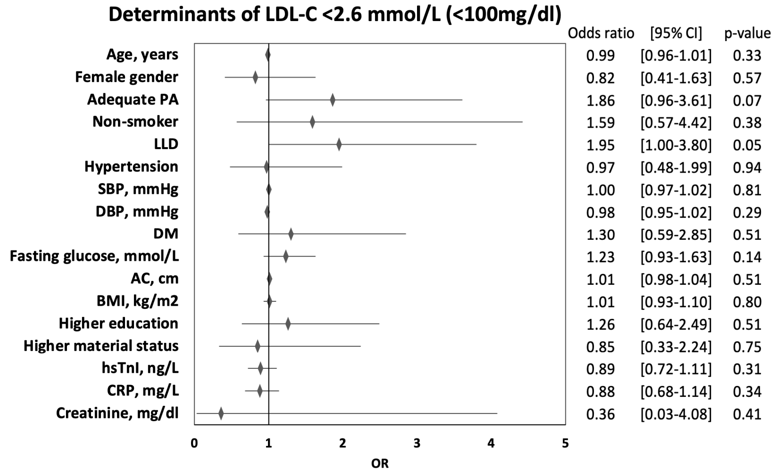

Figure S6. Determinants of LDL-C <2.6 mmol/L (<100 mg/dl) based on the results of the univariate analysis.

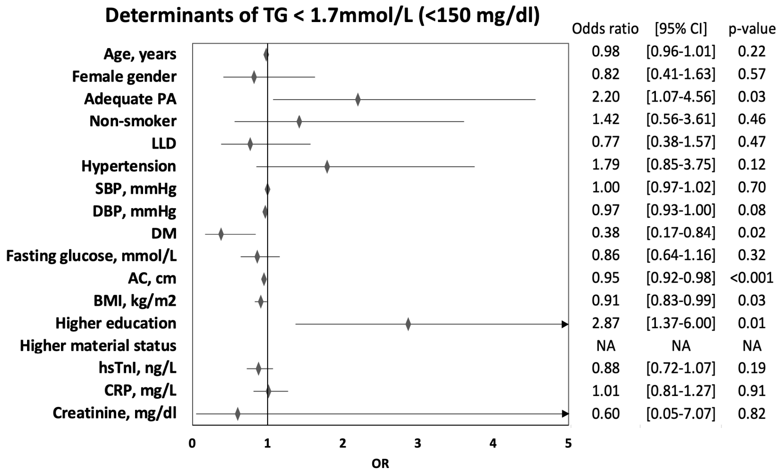

Figure S7. Determinants of TG <1.7 mmol/L (<150 mg/dl) based on the results of the univariate analysis.

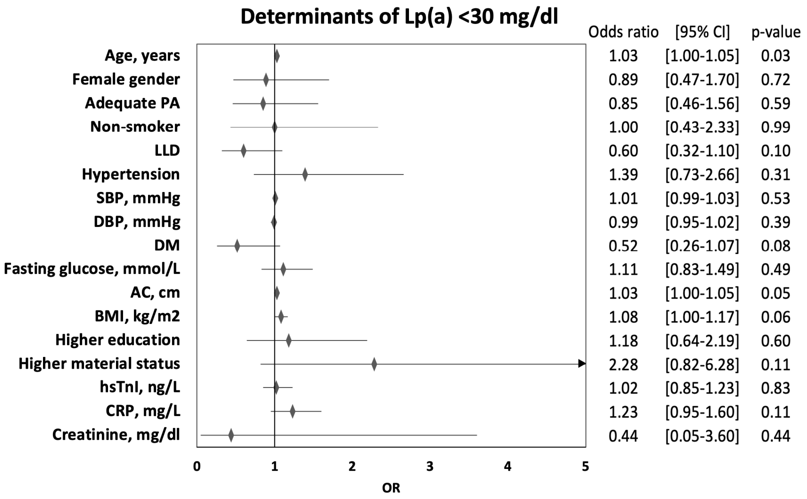

Figure S8. Determinants of Lp(a) <30 mg/dl based on the results of the univariate analysis.
